# Supplementary material for: The Cultural Brain Hypothesis: How culture drives brain expansion, sociality, and life history
Source: PLoS Comput Biol. 2018 Nov 8;14(11):e1006504. doi: 10.1371/journal.pcbi.1006504 (PMC6224031; doi:10.1371/journal.pcbi.1006504)
Supplement: S1 Supporting Information — (PDF) [file pcbi.1006504.s001.pdf]

Supplementary Material
to
The Cultural Brain Hypothesis: How culture drives brain expansion, sociality, and life history

Michael Muthukrishna, Michael Doebeli, Maciej Chudek & Joseph Henrich

Analytical Model

To explore the evolutionary adaptive dynamics of the CBH, we begin with individuals i represented by three continuous variables: brain size b_i, adaptive knowledge a_i, and reliance on social learning (over asocial learning; e.g. time spent), s_i. We will initially ignore the evolution of oblique learning, learning biases, and population structure, and assume that individuals using social learning use oblique learning and learning biases to hone in on the target individual with the most adaptive knowledge. We will relax this assumption in our simulation and allow oblique learning, learning biases, life history, and population structure to endogenously evolve. Table A is a handy key for the variables in our analytic model.

Table A. Variables for analytic model.

Variable	Description	Values
b_i	Brain size of individual i	$[0,\infty]$
a_i	Adaptive knowledge of individual i	$[0,b_i]$
s_i	Reliance on social learning (over asocial learning) of individual i	$[0,1]$
τ	Transmission fidelity	$[0,1]$
ζ	Asocial learning efficacy	$[0,1]$
r	Per capita birth rate	≥ 0
d	Per capita death rate	≥ 0
ϕ	Scaling coefficient used to scale relationship between adaptive knowledge and r	≥ 0
β	Scaling constant relating brain size to death rate (similar to κ in simulation)	≥ 0
λ	Death rate mitigation (e.g. richness of ecology) used to scale effect of a_i in reducing d	≥ 0
n	Increase in population size	$[-\infty,\infty]$

Individual i has two routes to acquire adaptive knowledge a_i: (1) through asocial (individual) learning as a function of their own brain size b_i and (2) through social learning as a function of the target model from whom they learn (a_m). The proportion of time or propensity to use social over asocial learning is given by s_i. Thus adaptive knowledge is given by:

$$a_i = s_i \cdot \tau \cdot a_m + (1 - s_i) \cdot \zeta \cdot b_i \quad (1)$$

Where τ is transmission fidelity (how well an individual can learn from a model), ζ is asocial learning efficacy (how effectively an individual can use their brain to figure things out), and a_m is the adaptive knowledge possessed by the individual in the parent generation from whom they are learning (e.g. model with maximal a or model with average a, etc).

The parameters τ and ζ in Equation 1 are abstractions of more complicated details covered in other work. By outsourcing the evolution of these features to other models, we can focus on the core of the CBH argument; i.e. how learning, brain size, knowledge, sociality, and life history are interconnected. Examples of this earlier work includes, Lewis and Laland [1] model of the relationship between transmission fidelity and the rate of trait loss, showing that sufficiently high transmission fidelity is necessary for cumulative culture, even more so than novel invention, incremental improvement, and recombination. Relatedly, building on work by Henrich [2], Mesoudi [3] models how increases in cumulative culture (driven by, for example, sociality) are more difficult for each generation to acquire. Thus, selection favors mechanisms to increase transmission fidelity. Muthukrishna and Henrich [4] discuss the many mechanisms to increase transmission fidelity as adaptive knowledge accumulates. Mechanisms such as explicit teaching may not be required in a small-scale society, but in a large-scale society, not only is explicit teaching required, but also formal institutionalized schooling from a variety of teachers. Thus, τ could include individuals' cognitive abilities [itself increased by culture; see 4], but also greater social tolerance, more interactions or opportunities for interaction, and some passive or active teaching by models, and so on [for more examples, see 4, 5, 6]. Transmission fidelity could be broken down into constraints and endogenous state variables for genetic, cultural, and social factors, as well as interactions between these (e.g. genes for sociality), but for the purposes of expressing our argument, here we capture all this with τ.

Similarly, our model relies on the idea that "bigger" brains will be better at solving novel problems, and figuring stuff out [7, 8]. As Deaner, et al. [7] analyses reveal, at least in primates, the best predictor of cognitive ability is overall brain size. But, as with transmission fidelity, many factors will influence individuals' ability to use their brains, such as constraints on time (for trial and error learning) or energy. These constraints are captured by ζ.

We take an evolutionary adaptive dynamics approach to find the evolutionary stable strategies (ESS) in our model. This approach involves assuming a monomorphic population and then looking at the "invasibility" of the population to a mutant (in variables of interest) with slightly different values. Appropriate to the dynamics we are interested in, this analytic method assumes mutations are small (i.e. we are not exploring competition between two vastly different groups).

Social Learning. To determine the average adaptive knowledge in a population that is monomorphic for resident genotype (s, b), we'll initially assume that genotype is fixed over the course of learning. We'll assume that the learning process leads to a distribution of adaptive knowledge values in the population and that individuals using social learning select a model using payoff-biased learning, choosing to learn from the model with the maximal possible value of adaptive knowledge a_{max} (i.e., they learn from the rare individual who has attained the maximal value). In the simulation, we will relax this assumption and allow oblique learning (learning from

non-genetic parents) and learning bias to evolve. Assuming individuals do learn from the best model when social learning, the mean adaptive knowledge in the population is given by:

$$\bar{a}(s,b) = s \cdot \tau \cdot a_{max} + (1-s) \cdot \zeta \cdot b \tag{2}$$

We further assume that the maximal adaptive knowledge is constrained by the brain size of the learner, such that $a_{max} = \nu b$, where $\nu > 0$ is some scaling parameter. As we shall see, the insights of the model are independent of the specific value of the ν scaling. Thus, Equation 2 becomes:

$$\bar{a}(s,b) = s \cdot \tau \cdot \nu \cdot b + (1-s) \cdot \zeta \cdot b \tag{2a}$$

We can now easily understand the adaptive dynamics of the social learning trait (s) assuming more adaptive knowledge has a higher payoff. For a given brain size (b), we simply compare $\tau\nu b$ and ζb: if $\tau\nu b > \zeta b$, then it pays to increase s as much as possible to maximize adaptive knowledge (i.e. $s \to 1$); conversely, if $\tau\nu b < \zeta b$, then it pays to decrease s as much as possible to maximize adaptive knowledge (i.e. $s \to 0$). This will be true as long as individuals have access to a range of models and are learning from the model with the greatest adaptive knowledge. Given these conditions, the key to reliance on social learning is the ability to learn with high fidelity and the key to reliance on asocial learning is the ability to efficiently use one's brain to learn by oneself. Further, if there is some limitation on accessing the model with the maximal adaptive knowledge, such as ineffective payoff biased learning making it difficult to identify who has the most adaptive knowledge or too small or disconnected a population for at least one individual to consistently reach this maximal value every generation, then the evolution of social learning is also going to depend on the maximal adaptive knowledge learners have access to. We explore these dynamics in the simulation model.

Brain Size. To determine the adaptive dynamics of brain size, we need an ecological model for monomorphic populations (i.e. for populations that consist of a single resident type (s,b)). To do this, we need to specify how the various traits affect the birth and death rates in the model. We use a logistic ecological model:

$$\frac{dN}{dt} = (r-d) \cdot N \tag{3}$$

Here N is population density, r is the per capita birth rate of the resident and d is the per capita death rate of the resident. Next, we specify the per capita birth rate (r) and death rate (d). We assume the birth rate r decreases with population size (density dependence influencing carrying capacity), but that that decrease is slower with increased adaptive knowledge (e.g. allowing you to support more offspring or outcompete competitors in access to mating opportunities). The birth rate (r) is given by:

$$\begin{aligned} r &= \rho\left(1 - \frac{N}{k_0 + k_1\bar{a}}\right) \\ &= \rho\left(1 - \frac{N}{k_0 + k_1(s \cdot \tau \cdot \nu \cdot b + (1-s) \cdot \zeta \cdot b)}\right) \end{aligned} \tag{4}$$

Where ρ is the maximal birth rate and that dependence leads to a linear decrease in the birth rate given by the second half of Equation 4. This linear decrease is assumed to be influenced by the mean adaptive knowledge $(\bar{a})$, such that more adaptive knowledge leads to a larger denominator, slowing the decrease with density dependence (allowing for a higher effective carrying capacity). k_0 and k_1 are positive parameters, which we set to 1, without loss of generality, in the following analyses.

We assume that a larger brain is more costly than a smaller brain in terms of death rate (e.g. higher calorie requirements), but that more adaptive knowledge lowers the death rate (e.g. finding food or evading predators). The death rate (d) is given by:

$$d = \beta \cdot b^n \cdot e^{-\lambda^a/_b} \quad (5)$$

This function assumes that the cost of brains scales up in a polynomial fashion (e.g. $n = 2$), but that the reduction in the death rate through adaptive knowledge is an exponential decay, where adaptive knowledge is bounded by brain size (i.e. $a \leq \nu b$). Here β scales the maximum brain size and λ scales the death rate reducing payoff to adaptive knowledge. The degree to which adaptive knowledge can offset brain size is determined by λ and the ratio of adaptive knowledge to brain size (adaptive knowledge is constrained by brain size regardless of learning mechanism and as brains grow, more knowledge is required to provide an equivalent offset). The λ parameter allows us to adjust the extent to which adaptive knowledge can offset the costs of brain size, where $\lambda = 0$ indicates no offset and increasing λ increases the probability of survival for a given adaptive knowledge and brain size. The λ parameter can be interpreted as how much adaptive knowledge one requires to unlock the fitness-enhancing advantages. For example, in a calorie-rich environment where only a little skill or knowledge is required to access calories (e.g. simply remembering food locations), λ would be high – a little bit of knowledge gives a large return. Conversely, in a calorie-poor environment where a lot of skills or knowledge are required to access fewer calories (e.g. food needs significant preparation before safe consumption), λ would be low. Note that this is a mechanical relationship between adaptive knowledge and probability of survival. Calorie availability is a potentially useful metaphor to think about the model in concrete term, but its is not the only interpretation of λ, which could be influenced any number of factors, including knowledge required to evade predators or avoid environmental hazards. We are also not directly saying anything about selection on cognition, brain size, or information use [9]. For example, high λ might allow for larger brains due to greater food availability for given food finding knowledge, but equally, when λ is low, there may be selection pressure for larger brains with more knowledge needed to acquire the more difficult to access food.

In the analytic model, the decrease to the death rate through adaptive knowledge becomes a constant since adaptive knowledge is a function of brain size (and parameters affecting learning efficiency), but although this will not affect the dynamics of the model, it will affect the final brain sizes. We fully explore this in the simulation.

Given a resident (s, b), the equilibrium population size of the resident is determined by the solution to Equation 3:

$$r(N^*) = d(b) \quad (6)$$

Since we know that $s \to 0$ when $\tau\nu < \zeta$ and $s \to 1$ when $\tau\nu > \zeta$, we can consider these two cases, asocial learners and social learners, separately and then compare the outcomes of these two regimes.

Asocial learners ($s = 0$)

To determine the adaptive dynamics of brain size, consider a mutant (designated by subscript "m") with brain b_m. This mutant's adaptive knowledge based on Equation 1 will be $a_m = \zeta b_m$, since $s = 0$. Using the same ecological assumptions as before for a mutant type b_m, and assuming the mutant is rare and growing (initially) in a resident population that is at its ecological equilibrium N^*, the per capita growth rate of the mutant, its invasion fitness, is:

$$
\begin{aligned}
f(b, b_{\mathrm{m}}) &= r_m(N^*) - d(b_m) \\
&= \rho\left(1 - \frac{N^*}{1 + a_m}\right) - \beta \cdot b_m^n \cdot e^{-\lambda^{a_m}/b_m} \\
&= \rho\left(1 - \frac{N^*}{1 + \zeta b_m}\right) - \beta \cdot b_m^n \cdot e^{-\lambda^{\zeta b_m}/b_m} \\
&= \rho\left(1 - \frac{N^*}{1 + \zeta b_m}\right) - \beta \cdot b_m^n \cdot e^{-\lambda\zeta}
\end{aligned} \tag{7}
$$

To examine the adaptive dynamics of brain size, we need to calculate the selection gradient by taking the derivative of the invasion fitness f with respect to the mutant trait b_m and evaluate this derivative at the resident value b. To calculate if these equilibria are stable, we will calculate the second derivative. If the second derivative is negative, then the value is a convergent stable ESS. For those unfamiliar with this approach, it may be helpful to use a physical analog—distance, speed, and acceleration (or more accurately, displacement, velocity, and acceleration). The derivative of distance over time (metres) is speed (metres per second). The second derivative (derivative of speed) is acceleration (metres per second per second). The adaptive dynamics approach is the equivalent of looking at when an object is stationary (i.e. speed—derivative of distance—is 0) and confirming that these "equilibria" stationary points are convergent by confirming that objects decelerate around these points (i.e. acceleration—second derivative—is negative). If the second derivative were positive, objects would increase speed and move away from this stationary point, or in the present case, there would be positive selection for mutants away from this equilibrium. Let us calculate the selection gradient for brain size:

$$
\frac{db}{dt} = \frac{\delta f}{\delta b_m}\Big|_{b_m = b} = \frac{\rho\zeta N^*}{(1 + \zeta b)^2} - n\beta e^{-\lambda\zeta} b^{n-1} \tag{8}
$$

From Equation 8 we can see that if $n > 1$, $db/dt < 0$ for large b and $db/dt > 0$ for small b, which suggests that there is some intermediate ESS value for brain size (b^*). It is straightforward to check that the second derivative of the invasion fitness function (Equation 8) with respect to the mutant trait and evaluated at the resident trait is always negative and therefore the singular strategy b^* is a CSS (i.e., a convergent stable ESS). This equilibrium brain value (i.e. when $db/dt = 0$) is difficult to solve for a generic polynomial n. To calculate a solution, we can select a reasonable polynomial (e.g. $n = 2$, which we use in the simulation) and solve for $db/dt = 0$. As long as brain size is positive, the relationship between brain size and the death rate will be superlinear and monotonous; our qualitative results should be robust to the specific polynomial used. Here is the equilibrium brain size for $n = 2$:

$$
b^* = \frac{-\beta + \sqrt{\beta^2 + 3\rho\zeta^2\beta e^{\lambda\zeta}}}{3\zeta\beta} \tag{9}
$$

We need to compare the equilibrium brain size among asocial learners expressed in Equation 9 with the equilibrium brain size among social learners, so let's now calculate the dynamics for social learners.

Social learners ($s = 1$)

To determine the adaptive dynamics of brain size, consider a mutant (designated by subscript "m") with brain b_m. This mutant's adaptive knowledge based on Equation 1 will be $a_m = \tau\nu b_m$, since

$s = 1$. Using the same ecological assumptions as before for a mutant type b_m, and assuming the mutant is rare and growing (initially) in a resident population that is at its ecological equilibrium N^*, the per capita growth rate of the mutant, its invasion fitness, is:

$$\begin{aligned} f(b_{\text{res}}, b_{\text{m}}) &= r_m(N^*) - d(b_m) \\ &= \rho\left(1 - \frac{N^*}{1 + a_m}\right) - \beta \cdot b_m^n \cdot e^{-\lambda^{a_m}/b_m} \\ &= \rho\left(1 - \frac{N^*}{1 + \tau\nu b_m}\right) - \beta \cdot b_m^n \cdot e^{-\lambda^{\tau\nu b_m}/b_m} \\ &= \rho\left(1 - \frac{N^*}{1 + \tau\nu b_m}\right) - \beta \cdot b_m^n \cdot e^{-\lambda\tau\nu} \end{aligned} \quad (10)$$

As before, to examine the adaptive dynamics of brain size, we need to calculate the selection gradient by taking the derivative of the invasion fitness f with respect to the mutant trait b_m and evaluate this derivative at the resident value b_{res}. To calculate if these equilibria are stable, we will calculate the second derivative. If the second derivative is negative, then the value is a convergent stable ESS. Let us calculate the selection gradient for the brain size of social learners:

$$\frac{db}{dt} = \frac{\delta f}{\delta b_m}\Big|_{b_m = b_{res}} = \frac{\rho\tau\nu N^*}{(1 + \tau\nu b)^2} - n\beta e^{-\lambda\tau\nu} b^{n-1} \quad (11)$$

As with asocial learners, from Equation 11 we can see that if $n > 1$, $db/dt < 0$ for large b and $db/dt > 0$ for small b, which suggests that there is some intermediate ESS value for brain size (b^*). As before, it is straightforward to check that the second derivative of the invasion fitness function (Equation 11) with respect to the mutant trait and evaluated at the resident trait is always negative and therefore the singular strategy b^* is a CSS (i.e., a convergent stable ESS). We can set $n = 2$ and calculate this equilibrium brain value (i.e. when $db/dt = 0$):

$$b^* = \frac{-\beta + \sqrt{\beta^2 + 3\rho\tau^2\nu^2\beta e^{\lambda\zeta}}}{3\tau\nu\beta} \quad (12)$$

Equation 12 is functionally similar to Equation 9, but the equilibrium brain size for asocial and social learners will be different. Moreover, since to enter the realm of social learning, $\tau\nu b > \zeta b$, social learners, *ceteris paribus*, will have larger equilibrium brain sizes than asocial learners. Note this prediction – that social learners will have larger brain sizes than asocial learners – is an outcome of the model, not an assumption. Moreover, transmission fidelity, asocial learning efficacy, and the payoff for adaptive knowledge (e.g. richness of the environment) are all going to affect the equilibrium brain size.

References

1. Lewis HM, Laland KN. Transmission fidelity is the key to the build-up of cumulative culture. Philosophical Transactions of the Royal Society B: Biological Sciences. 2012;367(1599):2171-80.
2. Henrich J. Demography and cultural evolution: how adaptive cultural processes can produce maladaptive losses: the Tasmanian case. American Antiquity. 2004;69(2):197-214.
3. Mesoudi A. Variable cultural acquisition costs constrain cumulative cultural evolution. PloS one. 2011;6(3):e18239.

4. Muthukrishna M, Henrich J. Innovation in the collective brain. Philosophical Transactions of the Royal Society of London B: Biological Sciences. 2016;371(1690). doi: 10.1098/rstb.2015.0192.
5. Dean LG, Kendal RL, Schapiro SJ, Thierry B, Laland KN. Identification of the social and cognitive processes underlying human cumulative culture. Science. 2012;335(6072):1114-8.
6. Whiten A, Erdal D. The human socio-cognitive niche and its evolutionary origins. Philosophical Transactions of the Royal Society of London B: Biological Sciences. 2012;367(1599):2119-29.
7. Deaner RO, Isler K, Burkart J, van Schaik C. Overall brain size, and not encephalization quotient, best predicts cognitive ability across non-human primates. Brain, Behavior and Evolution. 2007;70(2):115-24.
8. Sol D, Bacher S, Reader SM, Lefebvre L. Brain size predicts the success of mammal species introduced into novel environments. The American Naturalist. 2008;172(S1):S63-S71.
9. Dall SR, Giraldeau L-A, Olsson O, McNamara JM, Stephens DW. Information and its use by animals in evolutionary ecology. Trends in ecology & evolution. 2005;20(4):187-93.
10. Henrich J, Boyd R, Derex M, Kline MA, Mesoudi A, Muthukrishna M, et al. Understanding cumulative cultural evolution. Proceedings of the National Academy of Sciences. 2016. doi: 10.1073/pnas.1610005113.
